# Acidification changes affect the inflammasome in human nucleus pulposus cells

**DOI:** 10.1186/s12950-016-0137-0

**Published:** 2016-08-24

**Authors:** Frank J. Brand, Mahtab Forouzandeh, Harmanpreet Kaur, Francesco Travascio, Juan Pablo de Rivero Vaccari

**Affiliations:** 1 Department of Neurological Surgery, The Miami Project to Cure Paralysis, Miller School of Medicine, University of Miami, Miami, FL 33136 USA; 2 Biomechanics Research Laboratory, Department of Industrial Engineering, University of Miami, Coral Gables, FL 33146 USA; 3 Department of Neurological Surgery, Lois Pope LIFE Center, 1095 NW 14th Terrace, 3-25JJ, Miami, FL 33136-1060 USA

**Keywords:** Innate Immunity, Intervertebral disc degeneration, Caspase-1, Inflammasome, Inflammation

## Abstract

**Background:**

Interleukin (IL)-1β is involved in the pathology of intervertebral disc degeneration. Under normal conditions, IL-1β is present in cells in an inactive form (pro-IL-1β). However, under pathological conditions, pro-IL-1β is turned into its active form (IL-1β) by the inflammasome, a multi-protein complex of the innate immune response that activates caspase-1. Under conditions of degeneration, the disc experiences an environment of increased acidification. However, the implications of acidification on the innate immune response remain poorly explored.

**Methods:**

Here we have studied how pH changes in human nucleus pulposus cells affect inflammasome activation by immunoblot analysis of protein lysates obtained from nucleus pulposus cells that were exposed to different pH levels in culture.

**Results:**

In this study, we have found that in nucleus pulposus cells, with increased acidification, there was a decrease in inflammasome activation consistent with lower levels of active IL-1β. However, this effect at a pH of 6.5, the lowest pH level tested, was abrogated when cells were treated with IL-1β.

**Conclusions:**

Taken together, these findings suggest that the inflammatory response through IL-1β experienced by the human disc is not initiated in nucleus pulposus cells when the stimulus is acidification.

**Electronic supplementary material:**

The online version of this article (doi:10.1186/s12950-016-0137-0) contains supplementary material, which is available to authorized users.

## Background

Low back pain is a major pathological condition that affects approximately 84 % of the population at some point in their life [1]. Estimates indicate that in the United States, 25 % of the population suffers from low back or neck pain [2]. Accordingly, the societal costs associated with low back and neck pain exceeds $100 billion per year in the United States alone [3]. Intervertebral disc (IVD) degeneration (IDD) is believed to be a major contributor to back pain [4–7]. The etiology of IDD has been linked to genetic factors, aging or excessive manual labor [8]. All these factors contribute to increase inflammation, which eventually leads to increased degradation of aggrecan and collagen in the extracellular matrix of the disc [9].

Historically most studies looking at inflammation have focused on looking at professional immune cells as neutrophils and macrophages. However, recent studies indicate that cells that are not professionally considered as immune cells do play an important role on inflammation. This concept applies to cells as diverse as neurons [10–13] sperm cells [14, 15] or keratinocytes [16, 17]. Importantly, targeting inflammation in these cells has been shown to offer an important therapeutic potential. Thus in the context of IDD, it is important to understand the contribution of cells like NP or annulus fibrosus in regards to the inflammatory response in order to gain a better understanding of how inflammation contributes to IDD.

The inflammasome is a multi-protein complex in which caspase-1 is activated followed by processing of pro-interleukin (IL)-1β and pro-IL-18 into their respective active forms. The inflammasome has been previously shown to play a role in infections [18–20], metabolic syndromes [21, 22], autoimmune diseases [23–25] and injury [11, 13, 26–28]. A positive correlation between the degree of IDD and inflammasome content in the disc has also been previously reported [29].

The pro-inflammatory cytokines IL-1β and tumor necrosis factor (TNF) are two key cytokines that are involved in the pathology of IDD [9, 30–32] and degradation of the IVD [33, 34]. IL-1β is present in the cell in an inactive form as pro-IL-1β, and it relies on the inflammasome for its maturation into active IL-1β [35]. The inflammasome is a multi-protein complex comprised of a nod-like receptor (NLR) such as NLRP1 or NLRP3, as well as the adaptor protein apoptosis-associated speck-like protein containing a caspase recruitment domain (CARD) (ASC), and caspase-1 [36, 37]. In addition, X-linked inhibitor of apoptosis protein (XIAP) has been shown to maintain the inflammasome in an inhibited state [12, 13]. The inhibitory potential of XIAP is greatest in its full form (53 kDa) when compared to the cleaved fragment (23 kDa).

Three receptors have been identified to play a role upstream of inflammasome activation (caspase-1 cleavage). These include the pannexin-1 channel and the purinergic receptors P2X4 and P2X7 [11, 38]. These receptors rely on high extracellular potassium and adenosine tri-phosphate (ATP) for their activation, resulting in cleavage of caspase-1 [36, 39, 40]. However, the exact role that these receptors play on regulating inflammasome activation in IDD is under investigation [41–43]. Moreover, when activated, the inflammasome is not only responsible for triggering an innate immune response, but it is also involved in the cell death mechanism of pyroptosis [37, 44] that relies on the formation of ASC oligomers referred to as pyroptosomes [45].

The IVD is an avascular structure that is under constant metabolic demand; as a result, the IVD is constantly exposed to an environment of low oxygen, low glucose, and high lactic acid concentrations (acidic pH levels) [46]. It is estimated that the physiological pH level of the IVD is around 6.8 to 7.2, whereas in the degenerated state it can range between 6.6 and 6.3, or even lower in cases of severe degeneration [47, 48].

Two elements of IDD are acidification of the disc environment and inflammation. Previous studies have shown that metabolism and biosynthetic activity of disc cells markedly decrease at acidic pH levels [49, 50]. Importantly, the latter is in part mediated by the pro-inflammatory cytokine IL-1β. Therefore, in this study, we investigate the effects of acidification across different pH levels on the inflammatory response produced by human nucleus pulposus cells (HNPC) and regulated by the inflammasome, a major activator of the pro-inflammatory cytokine IL-1β.

## Methods

### Cell culture

Primary HNPC (Science Cell, Carlsbad, CA) cells were obtained from the spinal column (isolated from the nucleus pulposus of human intervertebral discs) of a male donor that did not present spine degenerative disease. Cells were grown in culture as a monolayer to 90 % confluency. Cells were passed two to three times before use in all experiments. HNPC were maintained in NPCM media (Science cell, Carlsbad, CA) containing 10 ml FBS (2 % FBS), 5 ml of NP cell growth supplement and 5 ml of penicillin/streptomycin solution in 500 ml of NPCM medium. Different groups of cells were grown in media at 3 different pH levels (7.4, 6.8 and 6.5) for 24 h. NPCM media at different pH levels were prepared by adding either sterilized HCl (1 mol/L) or NaOH (1 mol/L). pH levels were monitored with a Beckman 350 pH/Temp/mV Meter. Temperature was set at 36.5 °C and 5 % CO_2_. Sample size (N) presented in figure legends corresponds to the number of wells that received each treatment protocol.

### Immunoblotting

To determine levels of inflammasome signaling proteins, protein lysates were prepared and resolved by immunoblotting as described in [51]. Briefly, proteins were resolved in 10–20 % TGX Criterion precast gels (Bio-Rad, Hercules, CA), transferred to polyvinylidene difluoride membranes (Applied Biosystems, Foster City, CA) and placed in blocking buffer for 1 h (PBS, 0.1 % Tween 20, and 0.4 % I-Block (Applied Biosystems, Foster City, CA)). Membranes were incubated for 1 h with primary antibodies (1:1000) against caspase-1 (Novus Biologicals, Littleton, CO), ASC (Santa Cruz Biotechnology, Dallas, TX), caspase-5, (Millipore, Billerica, MA), XIAP (BD Transduction Laboratories, San Jose, CA), IL-1β (Cell Signaling, Billerica, MA), IL-18 (Millipore, Billerica, MA), NLRP1 (Millipore, Billerica, MA), NLRP2 (Abcam, Cambridge, MA), NLRP3 (Millipore, Billerica, MA), NLRC4 (Millipore, Billerica, MA), AIM2 (eBioscience, San Diego, CA), pannexin-1 (Invitrogen, Carlsbad, CA), P2X7 (Alamone Labs, Jerusalem, Israel) and P2X4 (Calbiochem, Billerica, MA). Membranes were then washed twice for 5 min in blocking buffer and incubated for 45 min with appropriate secondary horseradish peroxidase (HRP)-linked antibodies (1:1000; Cell Signaling, Billerica, MA). Proteins were visualized by chemiluminescence with a phototope-HRP detection kit (Cell Signaling, Billerica, MA). Band densities were quantified with UN-SCAN-IT software, and data were normalized to β-actin (1:5000; Sigma, St. Louis, MO).

### Simple Plex assay

To determine the protein concentration of released IL-1β and IL-18 at different pH levels, a Simple Plex assay was run using the Ella System (Protein System) according to manufacturer instructions. Briefly, 50 μl of diluted sample were loaded into separate wells of the cartridge, while 2 ml of washing buffer were loaded in the respective wells. The assay was then run by the Simple Plex Runner Software (Protein Simple) and analyzed by the Simple Plex Explorer (Protein Simple) based on analyte and lot specific factory curves. Results shown correspond to the mean of samples run in triplicates.

### Cell viability assay

To assess cell viability of HNPC at different pH levels, the CytoTox 96 Non-Radioactive Cytotoxicity Assay (Promega, Madison, WI) was used according to manufacturer’s instructions to evaluate the number of lysed cells. This assay measures the release of lactose dehydrogenase (LDH) that takes place upon cell lysis. For this experiment, the cell media was used to run the assay. The LDH release assay was carried after exposing cells to different concentration of IL-1β human recombinant protein (1, 5 and 10 ng/ml, Peprotech, Rocky Hill, NJ) for 24 h at a pH of 6.5.

### siRNA silencing

ASC/pycard stealth RNAi (Invitrogen, Carlsbad, CA) was used at a concentration of 40 nM. Cells were transfected with stealth RNAi for 3 days and then challenged with IL-1β human recombinant protein at a concentration of 10 ng/ml for 24 h at a pH of 6.5. The transfection protocol consisted of treating cells with Lipofectamine 3000 transfection reagent at a concentration of 40 nM according to manufacturer instructions. Stealth siRNA/Lipofectamine 3000 duplex was diluted on serum free medium (Opti-MEM I, Invitrogen, Carlsbad, CA).

### Statistical analysis

Statistical comparisons between groups were done using a one-way ANOVA followed by Holm-Sidak’s multiple comparisons test or a Tukey’s multiple comparisons test. The *p*-value of significance was set at *p* < 0.05.

## Results

### IL-1β and IL-18 expression in HNPC decreases at a pH of 6.5

**Fig. 1:**
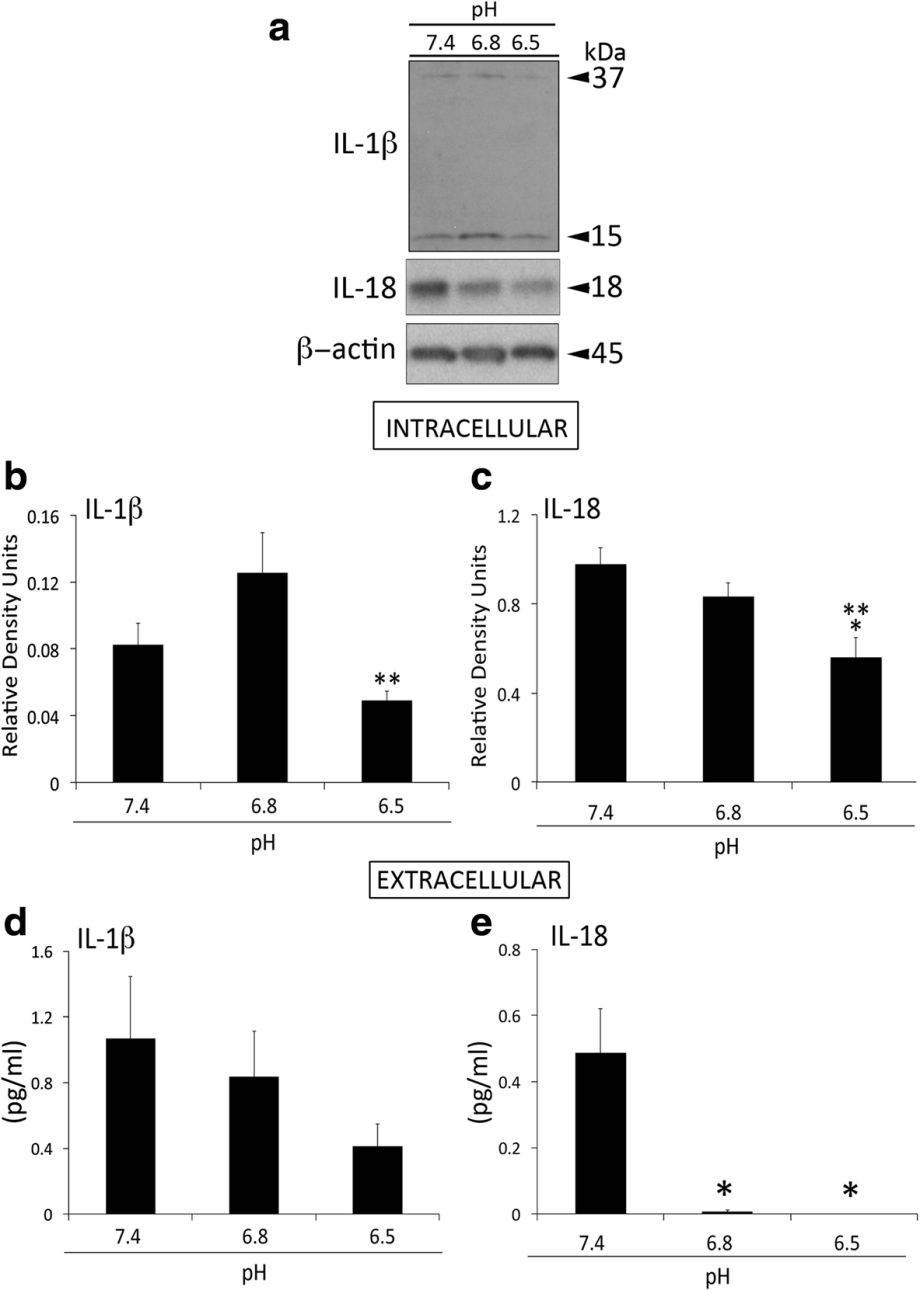
IL-1 cytokine expression is decreased at pathological pH. Representative immunoblot analysis of IL-1β and IL-18 at three different pH levels (7.4, 6.8 and 6.5) (**a**). Densitometric analysis corresponds to the active forms of IL-1β (15 kDa, **b**) and IL-18 (18 kDa, **c**). Data presented as mean+/−SEM. *N* = 6 wells per group. β-actin was used as an internal standard for protein loading control. Cell media were used to measure protein concentration of released IL-1β (**d**) and IL-18 (**e**). Data presented as mean+/−SEM. *N* = 5–6 wells per group. **p < 0.05 compared to 6.8 and *p < 0.05 compared to 7.4. Data on **a**, **b** and **c** were obtained from lysates measured by immunoblotting, and data from **d** to **e** correspond to supernatants measured by a Simple Plex Assay

IL-1β and IL-18 are two cytokines that rely on the inflammasome for their activation [36, 37]. IL-1β has been previously implicated in the pathology of IDD. Thus, to determine the effects of pH changes on the expression of IL-1β and IL-18 in HNPC (Fig. 1a), cells were grown in culture at a pH of 7.4 and then exposed to a pH of 6.8 and 6.5 for 24 h. Interestingly, the protein levels of IL-1β (Fig. 1b) and IL-18 (Fig. 1c) were decreased in HNPC at a pH of 6.5 when compared to a pH of 6.8. In addition, using a Simple Plex Assay (Ella, Protein Simple) we found that the amount of released IL-18 decreased at lower pH levels when compared to 7.4 (Fig. 1d, e). These findings indicate that, as a result of lowering the pH, the inflammatory response originating in HNPC is decreased.

### Inflammasome signaling protein expression in HNPC decreases at a pH of 6.5

**Fig. 2:**
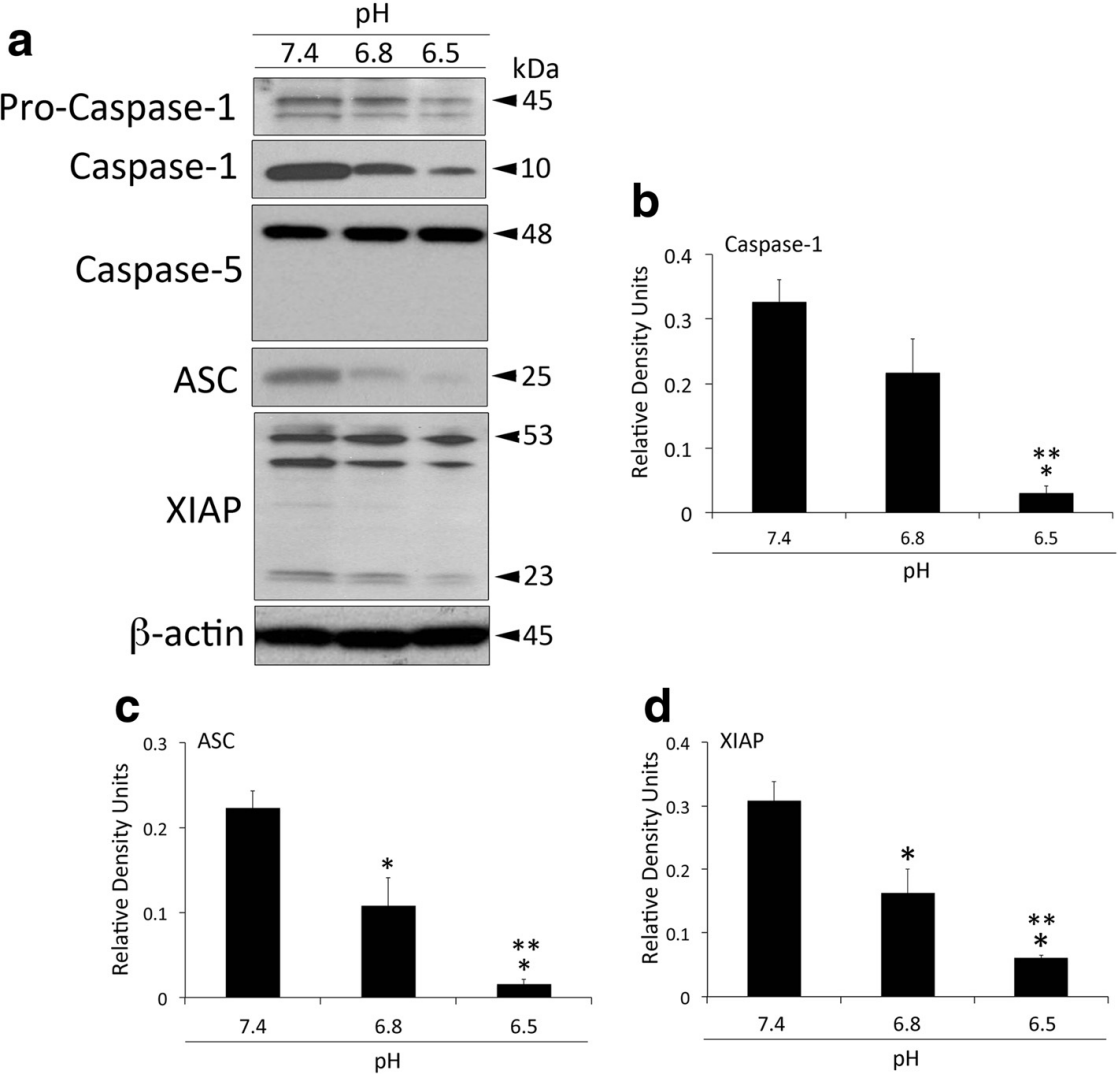
Inflammasome signaling protein expression decreases at pathological pH. Representative immunoblot analysis (**a**) of caspase-1, caspase-5, ASC and XIAP at three different pH levels (7.4, 6.8 and 6.5). Densitometric analysis corresponds to the active form of caspase-1 (10 kDa, **b**), ASC (**c**) and the cleaved fragment of XIAP (23 kDa, **d**). β-actin was used as an internal standard for protein loading control. Data presented as mean+/−SEM. **p < 0.05 compared to 6.8 and *p < 0.05 compared to 7.4. *N* = 6 wells per group

Considering the contribution of the inflammasome to the activation of the IL-1 cytokines IL-1β and IL-18 and the role that these cytokines play in IDD, we studied the protein expression levels of the inflammasome proteins caspase-1, caspase-5, ASC and XIAP (Fig. 2a). Of these proteins, caspase-1 (Fig. 2b), ASC (Fig. 2c) and XIAP (Fig. 2d) expression was decreased when acidification increased. There was no difference in caspase-5 (another inflammatory caspase present in humans) expression amongst the different pH levels that were tested in this study (Fig. 2a). These data are consistent with our findings of decreased IL-1β and IL-18 expression at this pH.

### NLRP1 and NLRP3 expressions in HNPC decrease at pathological levels of pH

**Fig. 3:**
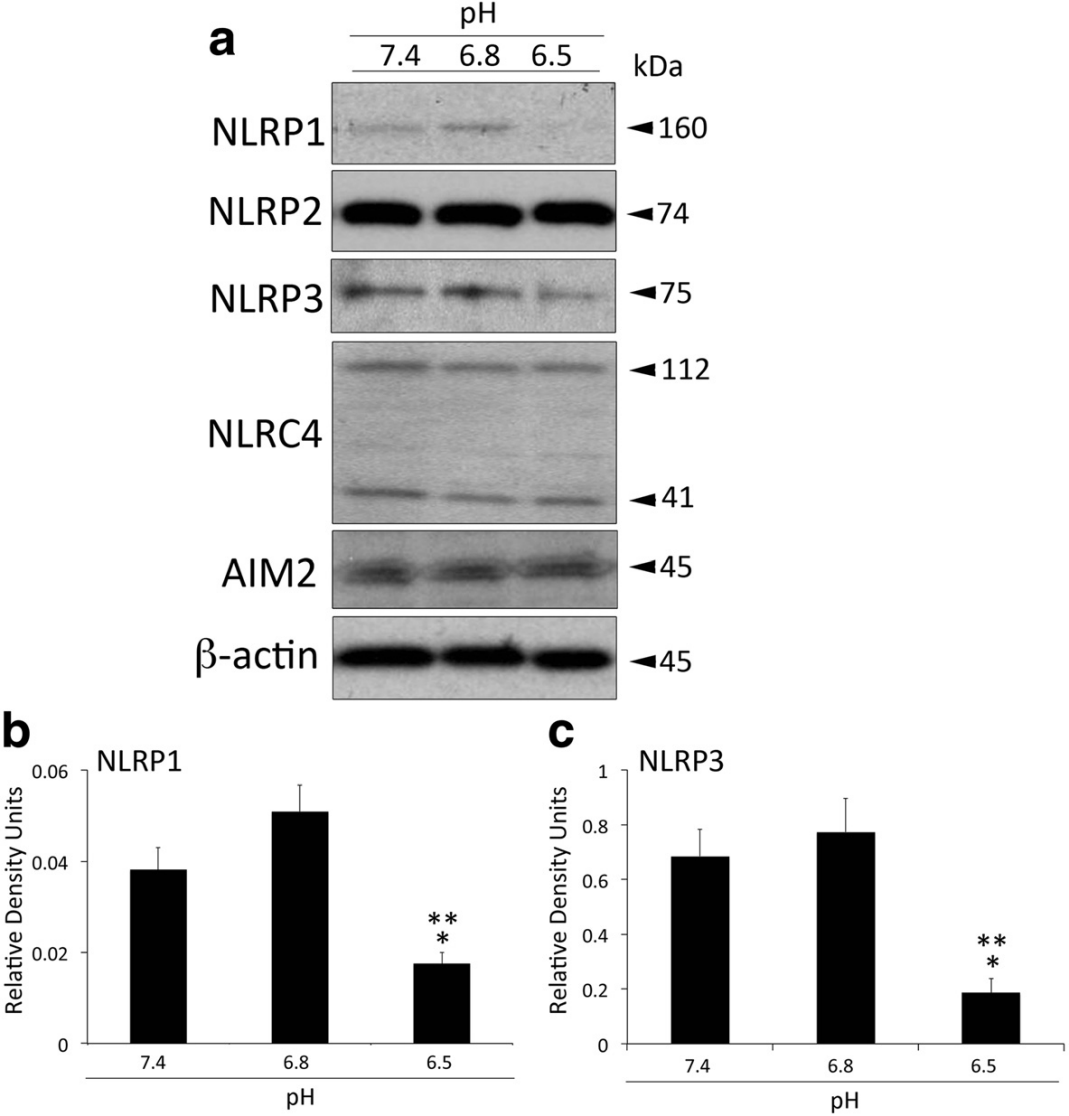
NLRP1 and NLRP3 protein expression is decreased at pathological pH. Representative immunoblot analysis (**a**) of NLRP1, NLRP2, NLRP3, NLRC4 and AIM2 at three different pH levels (7.4, 6.8 and 6.5). Densitometric analysis corresponds to NLRP1 (**b**) and NLRP3 (**c**). β-actin was used as an internal standard for protein loading control. Data presented as mean+/−SEM. **p < 0.05 compared to 6.8 and *p < 0.05 compared to 7.4. *N* = 6 wells per group

Inflammasomes are comprised of either a NOD-like receptor (NLR) or an AIM-2-like receptor (ALR) [36, 40]. To investigate the protein expression pattern of NLRs and ALR at different pH levels in HNPC, we immunoblotted for NLRP1, NLRP2, NLRP3, NLRC4 and AIM2 (Fig. 3a). At a pH of 6.5, NLRP1 (Fig. 3b) and NLRP3 (Fig. 3c) expressions were decreased; whereas NLRP2, NLRC4 and AIM2 protein levels were not affected (Fig. 3a).

### Inflammasome signaling receptor expression in HNPC decreases at a pH of 6. 5

**Fig. 4:**
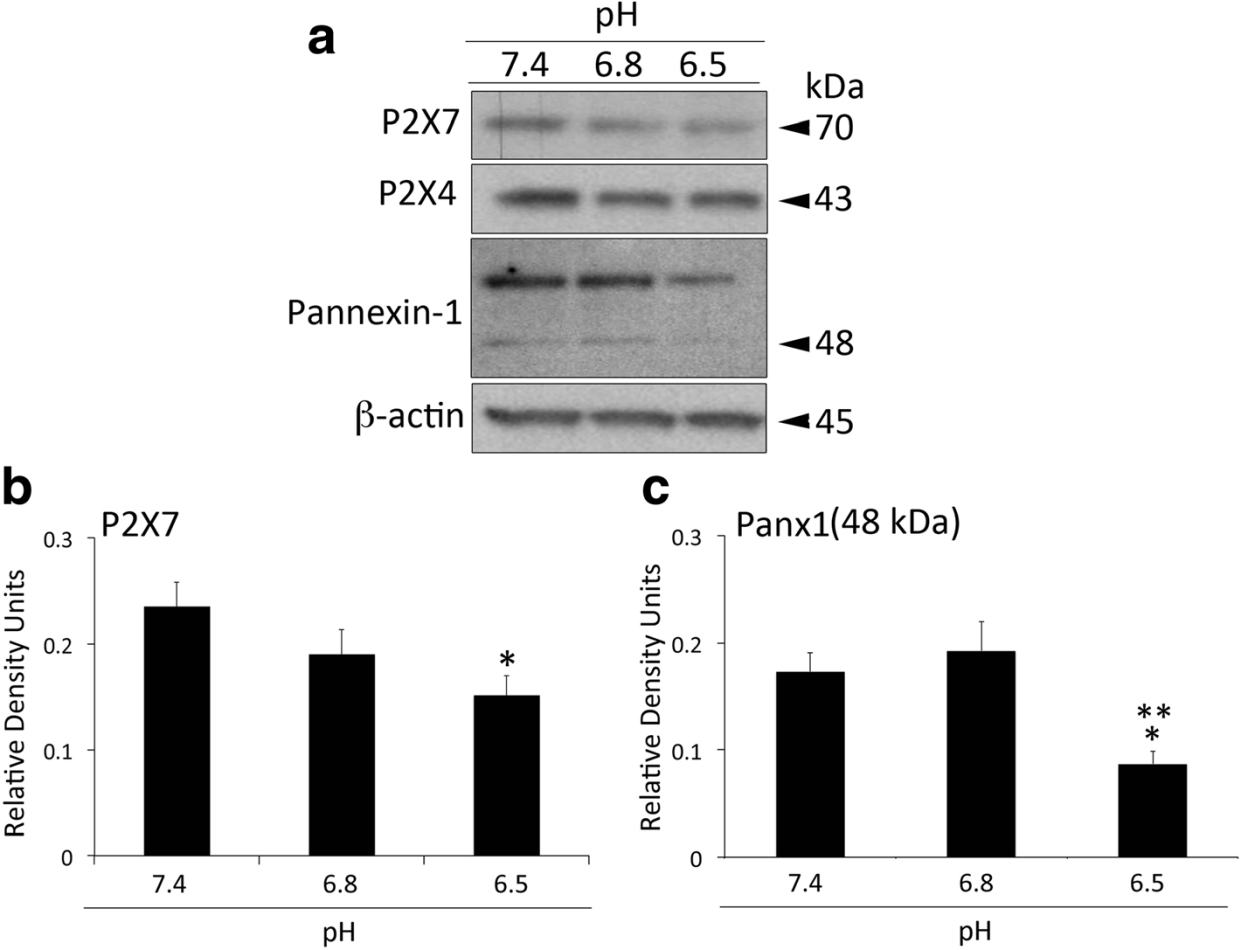
Pannexin-1 protein expression is decreased at pathological pH. Representative immunoblot analysis (**a**) of P2X7, P2X4 and pannexin-1 at three different pH levels (7.4, 6.8 and 6.5). Densitometric analysis corresponds to P2X7 (**b**) and pannexin-1 (Panx1) (**c**). β-actin was used as an internal standard for protein loading control. Data presented as mean+/−SEM. **p < 0.05 compared to 6.8 and *p < 0.05 compared to 7.4. *N* = 6 wells per group

We have previously identified P2X4 [11], P2X7 and pannexin-1 [38, 52] as receptors involved in the activation of the inflammasome. To test if the expression of these receptors is affected by different pH levels, we immunoblotted for these receptor proteins in lysates of HNPC (Fig. 4a). When compared to a pH of 6.8, pannexin-1 (Fig. 4c) was the only protein of these receptors that was significantly decreased. Whereas P2X7 levels were only significantly affected when compared to those attained at a pH of 7.4 (Fig. 4b). Whether pannexin-1 activity is affected by this change in pH is yet to be determined.

### pH changes affect HNPC viability

**Fig. 5:**
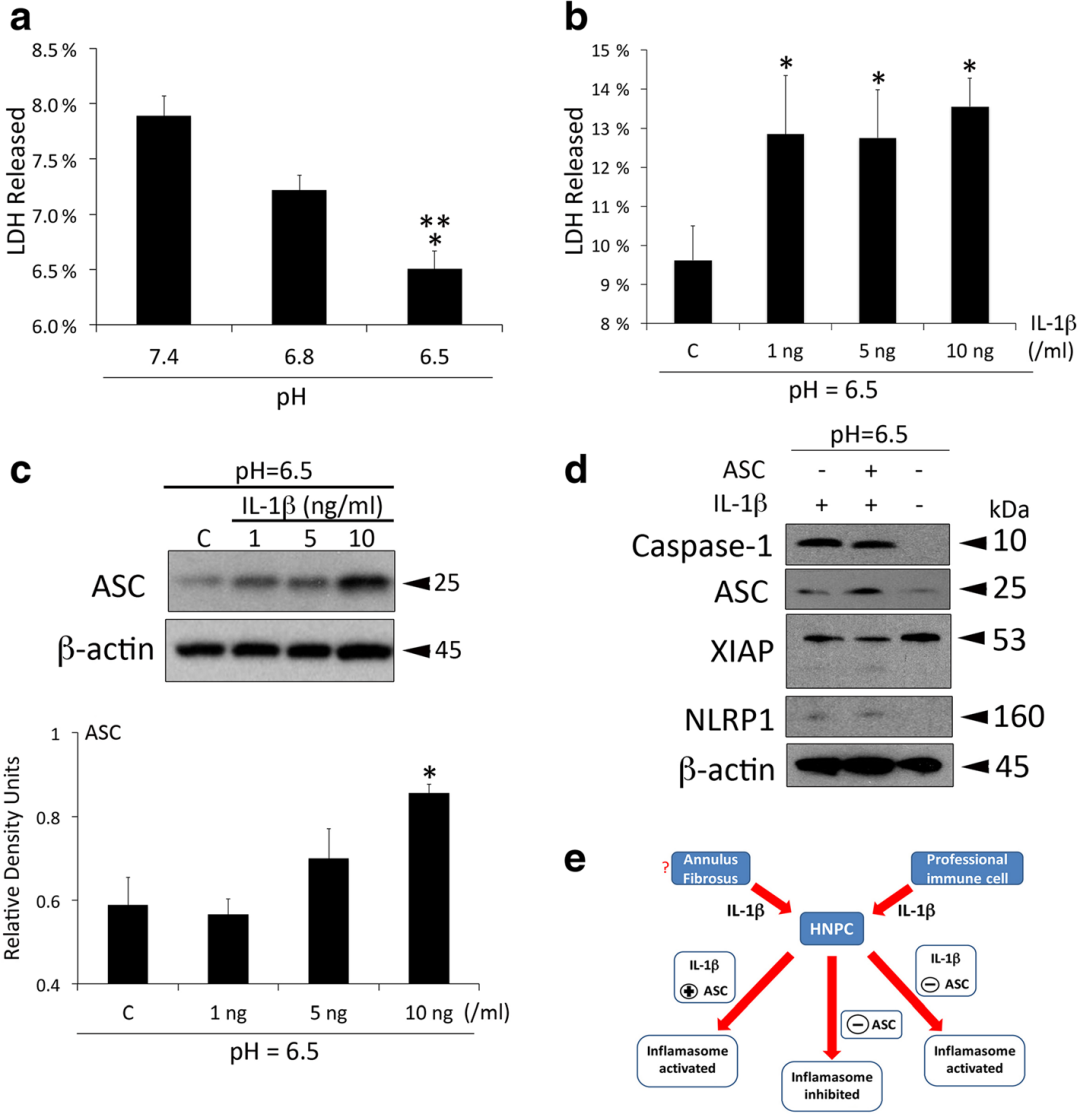
Exogenous IL-1β contributes to the inflammatory response in HNPC. Cells were grown at 3 different pH levels (7.4, 6.8, 6.5) for 24 h and the media was used to run a lactose dehydrogenase (LDH) release assay. Data presented as mean+/−SEM. **p < 0.05 compared to 6.8 and *p < 0.05 compared to 7.4. *N* = 6 wells per group (**a**). Cells were then grown at a pH of 6.5 and treated with human recombinant protein to IL-1β at different concentrations (1, 5, 10 ng/ml) (**b**). Representative immunoblot analysis of ASC protein expression in HNPC treated with different concentrations of IL-1β (1, 5, 10 ng/ml) for 24 h at a pH of 6.5 (**c**). β-actin was used as an internal standard for protein loading control. Data presented as mean+/−SEM. *p < 0.05 compared to control. *N* = 6 wells per group. Silencing of ASC/pycard by siRNA in HNPC resulted in increased caspase-1 and NLRP1 protein expression as well as increased XIAP cleavage when cells were treated with 10 ng/ml of human recombinant IL-1β protein at a pH of 6.5. (+) indicates ASC/pycard was not silenced and (−) indicates ASC was silenced (**d**). Representative model of the effects of ASC silencing and exposure to IL-1β on inflammasome activation in HNPC (**e**)

The metabolic activity of HNPC has been shown to decrease at lower pH levels [38, 52]. In this study we show that at a pH of 6.5, the levels of lactate dehydrogenase (LDH) release were also decreased (Fig. 5a). Interestingly, when the human IL-1β recombinant protein was added, the levels of LDH released were increased when compared to the untreated control group (Fig. 5b). These findings indicate that at lower pH levels, there is less cellular damage (less LDH released), but once IL-1β enters the system, then there is an increase in cell death/lysis. Moreover, exposure of HNPC to IL-1β also increased the expression of ASC, a key adaptor protein involved in the activation of the inflammasome (Fig. 5c). These findings indicate that there is a decrease in cell damage associated with lower acidity levels; however, IL-1β exposure overrides these effects in HNPC.

### IL-1β contributes to the inflammatory response in HNPC independently of the inflammasome signaling protein ASC

The basic components of the inflammasome are caspase-1 and a NOD-like receptor (NLR) protein such as NLRP1 [36, 40, 53]. ASC may or may not be present and when present, it is thought to be an enhancer of inflammasome activation. Moreover, ASC is a key protein involved in the activation of the inflammasome, which can be used as a therapeutic target against inflammation [12, 13, 54]. Since ASC expression responds to increased levels of IL-1β we then wanted to see the effects of IL-1β on the expression of other inflammasome proteins and whether these changes in protein expression were dependent on the inflammasome adaptor protein ASC. Therefore, to determine the role of ASC on the activation of the inflammasome, we carried out a gene silencing experiment against ASC/pycard and exposed cells to 10 ng of human IL-1β recombinant protein for 24 h. This concentration of IL-1β is consistent with previous studies showing that 10 ng/ml of IL-1 contributes to the pathogenesis of IDD [55]. Taken together, our findings indicate that when ASC was knocked-down, the expression of the inflammasome proteins cleaved caspase-1, NLRP1 and cleaved XIAP remained low. Interestingly, even when the protein levels of ASC were reduced, delivery of IL-1β recombinant protein was able to stimulate the production of cleaved caspase-1, ASC, NLRP1 and cleaved XIAP (Fig. 5d and Additional file 1: Figure S1), indicating that exogenous IL-1β has the potential to exacerbate the inflammatory response experienced by degenerating HNPC even in the absence of inflammasome activation, which is consistent with the pro-inflammatory role of IL-1β, further suggesting that exogenous IL-1β has the potential to exacerbate the inflammatory response experienced by degenerating HNPC independent of inflammasome activation (Fig. 5e).

## Discussion

Inflammation, a key contributor to the degeneration of the disc, is in part mediated by IL-1β [56]. To date, how the innate immune response regulates inflammation in the disc remains poorly understood. In the process of IDD, as the severity of degeneration increases, disc acidity also increases [47, 48]. Therefore, in this study we have tested how the inflammasome, a component of the innate immune response, regulates IL-1β production [48] at different pH levels.

In the process of IDD, disc cells must adjust to the balance between catabolic and anabolic activities in order to preserve the integrity of the extracellular matrix of the IVD. Acidification (i.e., decrease in pH levels) of the extracellular matrix (ECM) interferes with protein and proteoglycan synthesis in the IVD [50]. Accordingly, several factors contribute to the acidification of the discs’ ECM. First, glycosaminoglycans (GAG) in the disc carry a high negative charge due to the presence of carboxylate (COO^−−^) and sulfate (SO_4_^2−^), which attract Na^+^, K^+^ and H^+^ ions. As a result, the H^+^ concentration tends to be about 0.5 pH units lower than the surrounding serum or synovial fluid [57, 58]. Second, due to mechanical loading, the disc undergoes fluid expulsion. Once the fluid leaves the disc, the GAG concentration increases, thus the H^+^ concentration also increases, resulting in tissue acidification [59]. Third, the anaerobic glycolysis that takes place in the disc results in high levels of lactate [60]. Lactate diffusion across the disc is slow, resulting in increased acidity in the ECM of both NP and annulus fibrosus [61]; and fourth, the disc has a Na^+^-H^+^ exchanger that keeps the pH constant until lactate metabolism or transport is impaired by the degenerative process [62]. During IDD, blood supply to the disc is impaired, and the associated decrease in nutrient supply leads to lower pH levels [63]. Another contributor to the low pH levels in IDD is the production of inflammatory cytokines such as IL-1β and TNF, which have been shown to increase lactate production, and as a result, lower the pH and increase production of matrix metalloproteinases (MMP) [46, 61, 64, 65].

An involvement of the innate immune response through toll-like receptor (TLR) stimulation in IVD has recently been identified. In that study lipopolysaccharide (LPS) was used to stimulate TLR4 [66]. However, LPS is a ligand that can activate several immune complexes in addition to TLR4 [67], so it is possible that other TLRs may be activated in IVD. Moreover, since LPS is present during infections, further studies are needed to identify what danger/damage-associated molecular pattern activates pattern recognition receptors such as TLRs or the NLRs that form the inflammasome in IDD.

In addition, the NLRP3 inflammasome has been implicated in the pathology of IDD. Accordingly, higher levels of NLRP3 inflammasome proteins correlate with advanced grades of degenerated discs in humans [29]. We have previously shown that the NLRP1 inflammasome is active in neurons of the spinal cord after injury [13]. This neuronal NLRP1 inflammasome is comprised of caspase-1, caspase-11 (in rodents, and caspase-5 in humans), ASC and XIAP [13]. In the HNPC, we have found that the expression of the inflammasome proteins NLRP1, NLRP3, caspase-1 and ASC is decreased at a pH of 6.5. These data further suggest that the NLRP1 and/or NLRP3 inflammasomes are altered in the process of IDD. Furthermore, at a decreased pH of 6.5 we have found lower levels of cleaved XIAP, an inhibitor of the inflammasome. The inhibitory potential of XIAP is greatest when it is present in the full form (53 kDa) when compared to its cleaved fragments (23 kDa). Thus our data indicate that XIAP cleavage is decreased at lower pH levels, indicating a higher inhibitory potential on the inflammasome at a pH of 6.5, which is consistent with lower levels of cleaved caspase-1. Moreover, lower levels of cleaved (active) caspase-1 are consistent with decreased processing of pro-IL-1β (inactive) into IL-1β (active), thus signifying decreased inflammasome-1 activity.

Moreover, in the central nervous system, inhibition of the inflammasome by targeting the adaptor protein ASC results in improved histopathological and functional outcomes by decreasing inflammasome activation and IL-1β processing [12, 13]. For this reason, we targeted ASC using a siRNA approach and identified that, decreasing the expression of ASC, also affected the expression of caspase-1, cleaved XIAP and NLRP1. However, addition of the human recombinant IL-1β protein to the system resulted in increased expression of caspase-1 in HNPC, suggesting that the inflammatory response experienced by HNPC in IDD could originate in a different cell type like annulus fibrosus cells or even dorsal root ganglia or infiltrated inflammatory cells such as neutrophils. However, the effects described in this study on inflammasome regulation in HNPC are limited to pH changes as the insult. Therefore, the effects of other stimuli associated with IDD such as glucose changes or oxygen changes may contribute to the increase in IL-1β that can originate in NPC and that characterize IDD. Current studies are underway looking at inflammasome regulation as a result of these other physiological perturbations.

Three receptors have been identified with the activation of the inflammasome. These are the purinergic receptors P2X4 and P2X7 as well as the pannexin-1 channel [11, 38, 52]. In this study, we show that pannexin-1 expression was decreased at a pH of 6.5 when compared to 6.8 and 7.4. Pannexin-1 is involved in the activation of the inflammasome and the processing of IL-1β [38, 52]. Our findings showing lower levels of pannexin-1 expression at lower pH levels are consistent with our data indicating decreased caspase-1/inflammasome activation and decreased processing of IL-1β and IL-18 into their active forms. Thus, considering previous findings on the regulation of the inflammasome by pannexin-1, it is possible that in NP cells, pannexin-1 may act as an upstream regulator of the inflammasome.

We also detected a significant change in protein expression when comparing P2X7 at pH levels of 7.4 and 6.5. It is known that activation of P2X7 signaling results in lower cytosolic pH levels by affecting the intracellular proton balance [68, 69]. Nevertheless, extracellular acidification has also been shown to inhibit P2X7 activation [70]. In addition, extracellular ATP, which activates purinergic receptors, promotes extracellular matrix biosynthesis, whereas intracellular ATP promotes production of IVD cells [71].

In monocytes, acidic extracellular pH activates the inflammasome by a process associated with increased synthesis of pro-IL-1β [72]. Similarly, acidic pH changes activate the NLRP3 inflammasome in macrophages in response to LPS [73]. As a result, it has been suggested that extracellular acidification acts as an alarmin that triggers the innate immune response through the inflammasome [73]. In contrast, it is also possible that extracellular acidosis inhibits IL-1β-dependent innate immune activation following stimulation of purinergic receptors with ATP. This probably takes place as an attempt to dampen an exacerbated innate immune response [74], which is consistent with our findings showing decreased inflammasome activation at pH levels of 6.5. In this study, the protein expression levels correspond to the active form of IL-1β and IL-18. For this reason, we referred to these proteins as active.

Importantly, we have chosen pH levels considering the normal IVD extracellular environment, and the pH level of 6.5 that is attained during moderate to severe IDD. Accordingly, we do expect that at even lower pH levels (extreme degeneration, 6.3) the observed effects in this study would be even more pronounced.

Despite increased cell viability and decreased activation of the inflammasome as well as activation of the IL-1 cytokines IL-1β and IL-18 at a pH of 6.5, it is well known that IL-1β is increased in degenerating IVD. In contrast, here we show that inflammasome activation is decreased at the lower pH of 6.5 when compared to the physiological pH of most tissue fluids (7.4) as well as the more acidic environment in which IVD cells are found (6.8). The protein levels of caspase-1, NLRP1, NLRP3, ASC and cleaved XIAP as well as IL-1β and IL-18 were decreased at more acidic pH levels. These data are consistent with the findings of Razaq who showed that at lower pH, the production of ECM proteins was inhibited, yet the production of MMP was not decreased at lower pH levels. Therefore, the proteins responsible for maintaining the integrity of the disc are decreased while the proteins responsible for degrading the disc remain active, thus facilitating the degenerative process [48]. We suggest that at lower pH levels, the response of NP cells is to shut down the inflammatory machinery in order to prevent the burden of degeneration that is associated with inflammation. However, NP cells are still responsive to the effects of IL-1β originating from different cell types, which results in exacerbated inflammation. Accordingly, when we delivered IL-1β to HNPC following silencing of ASC, HNPC showed increased expression of caspase-1, cleaved XIAP and the NLR protein NLRP1. It is possible that the inflammasome in HNPC is needed to mount an inflammatory response in events such as infections or when the stimulus for damage is other than pH changes. However, here we focused on the physiology of HNPC when only the pH is altered. Under this scenario, when the pH is low and ASC is silenced, then caspase-1 is not activated, yet HNPC still remain able to respond to IL-1β from other sources, thus HNPC are still able to produce an inflammatory response that is ASC-independent but caspase-1-dependent upon stimulation with IL-1β. In addition, since ASC is considered an enhancer of the inflammatory response, it is possible that in the absence of ASC the caspase-1-dependent inflammatory response is milder than in the presence of ASC. Taken together, this would indicate that under low pH levels, the source of IL-1β that affects NPC during degeneration is other than NPC themselves. Whether, the source of inflammation is leukocytes, annulus fibrosus cells or originating from dorsal root ganglia is under investigation (Fig. 5e). In this regard, it has been suggested that IVD cells are both the effector cells and the target cells of the inflammatory response [75]. Accordingly, infiltration of professional inflammatory cells into the disc seems to be a secondary inflammatory event in which inflammatory cells are able to penetrate into the disc tissue, but for this penetration to be possible, the disc should already have signs of degeneration. Future studies will look at the mechanism of how pH changes affect inflammasome protein expression. However, it is possible that this regulation occurs by modulating NF-kB, a key transcriptional regulator of inflammasome signaling. In addition, it is important to consider that these studies were carried on cells that were not isolated from degenerated discs. Therefore, future studies need to focus on how pH changes affect the inflammasome in cells obtained from different grades of degenerated discs. However, the different levels of pH used in this study aimed to reflect different levels of disc degeneration.

## Conclusions

The data in this study indicate that under increased acidification conditions (6.5 pH) such as those experienced during severe IDD, the main response of the NP cells is to decrease its production of IL-1β by decreasing inflammasome activation. Accordingly, we suggest that the inflammation experienced by the NP originates either from the annulus fibrosus or from infiltrated cells such as leukocytes, or even the dorsal root ganglia, which we are investigating at this time. However, other stimuli besides increased acidification, like altered oxygenation or altered glucose levels, may also be the triggers for the increased IL-1β levels that are found in the NP during IDD.

## Additional file

Additional file 1: Figure S1. Immunoblot of ASC following gene silencing procedures for ASC using siRNA (si) against ASC as well as the scrambled siRNA control (scr), indicating that the scrambled control did not affect ASC expression. (JPG 110 kb)

## Abbreviations

ALR: AIM-2-like receptor
ASC: Apoptosis-associated speck-like protein containing a caspase recruitment domain
ATP: Adenosine tri-phosphate
ECM: Extracellular matrix
HNPC: Human nucleus pulposus cells
IDD: Intervertebral disc degeneration
IL: Interleukin
IVD: Intervertebral disc
LDH: Lactose dehydrogenase
LPS: Lipopolysaccharide
MMP: Matrix metalloproteinases
NLR: NOD-like receptor
TLR: Toll-like receptor
TNF: Tumor necrosis factor
XIAP: X-linked inhibitor of apoptosis protein
