# Supplementary figures and images for: Acidification changes affect the inflammasome in human nucleus pulposus cells
Source: J Inflamm (Lond). 2016 Aug 24;13(1):29. doi: 10.1186/s12950-016-0137-0 (PMC4997758; doi:10.1186/s12950-016-0137-0)

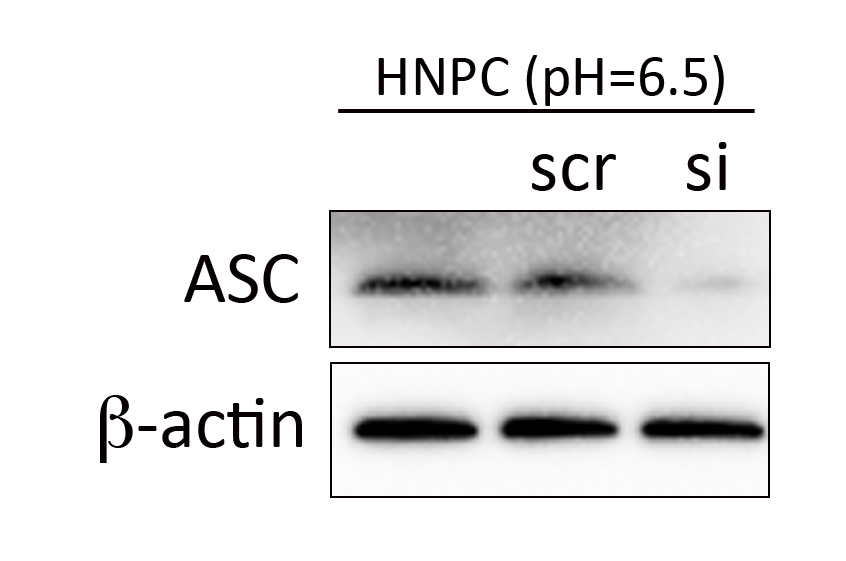

Supplement: Additional file 1: Figure S1. — Immunoblot of ASC following gene silencing procedures for ASC using siRNA (si) against ASC as well as the scrambled siRNA control (scr), indicating that the scrambled control did not affect ASC expression. (JPG 110 kb) [file 12950_2016_137_MOESM1_ESM.jpg]
